# A Literature Review on the Role of the Invasive *Aedes albopictus* in the Transmission of Avian Malaria Parasites

**DOI:** 10.3390/ani14142019

**Published:** 2024-07-09

**Authors:** Jesús Veiga, Mario Garrido, Marta Garrigós, Carolina R. F. Chagas, Josué Martínez-de la Puente

**Affiliations:** 1 Departamento de Biología de la Conservación y Cambio Global, Estación Biológica de Doñana (EBD, CSIC), 41092 Sevilla, Spain; 2 Department of Parasitology, Faculty of Pharmacy, University of Granada, 18011 Granada, Spain; m.garrido@ugr.es; 3 Nature Research Centre, Akademijos St. 2, 08412 Vilnius, Lithuania; carolina.chagas@gamtc.lt; 4 Ciber de Epidemiología y Salud Pública (CIBERESP), 28029 Madrid, Spain

**Keywords:** Asian tiger mosquito, avian *Plasmodium*, blood parasites, haemosporidians, invasive species, insect vectors

## Abstract

**Simple Summary:**

*Aedes albopictus* is an invasive mosquito species with a currently broad distribution range. In this article, we address the role of this mosquito species in the transmission of avian malaria parasites of the genus *Plasmodium*. To do that, we review the literature and compile existing information on *Ae. albopictus*–avian *Plasmodium* interactions to consider the potential impact of its expansion on avian malaria epidemiology. The findings suggest that while experimental infection of certain *Plasmodium* species is feasible, the occurrence of avian *Plasmodium* in wild Asian tiger mosquitoes is rare. Mosquitoes’ preference for mammals as hosts and their relatively low susceptibility to infection may explain these results.

**Abstract:**

The Asian tiger mosquito (*Aedes albopictus*) is an invasive mosquito species with a global distribution. This species has populations established in most continents, being considered one of the 100 most dangerous invasive species. Invasions of mosquitoes such as *Ae. albopictus* could facilitate local transmission of pathogens, impacting the epidemiology of some mosquito-borne diseases. *Aedes albopictus* is a vector of several pathogens affecting humans, including viruses such as dengue virus, Zika virus and Chikungunya virus, as well as parasites such as *Dirofilaria*. However, information about its competence for the transmission of parasites affecting wildlife, such as avian malaria parasites, is limited. In this literature review, we aim to explore the current knowledge about the relationships between *Ae. albopictus* and avian *Plasmodium* to understand the role of this mosquito species in avian malaria transmission. The prevalence of avian *Plasmodium* in field-collected *Ae. albopictus* is generally low, although studies have been conducted in a small proportion of the affected countries. In addition, the competence of *Ae. albopictus* for the transmission of avian malaria parasites has been only proved for certain *Plasmodium* morphospecies under laboratory conditions. Therefore, *Ae. albopictus* may play a minor role in avian *Plasmodium* transmission in the wild, likely due to its mammal-biased blood-feeding pattern and its reduced competence for the development of different avian *Plasmodium*. However, further studies considering other avian *Plasmodium* species and lineages circulating under natural conditions should be carried out to properly assess the vectorial role of *Ae. albopictus* for the *Plasmodium* species naturally circulating in its distribution range.

## 1. Introduction

*Aedes* (*Stegomyia*) *albopictus*, commonly known as the Asian tiger mosquito, is an invasive species native to southeast Asia covering from Japan and China to tropical countries such as Malaysia and Singapore [1]. This invasive mosquito species has a global distribution with established populations in most continents, including North and South America, Africa, Asia, and Europe [2]. Overall, the species has been recorded in at least 126 countries worldwide [3], and it is expected that its distribution range will continue to increase [4]. It is forecast that it will colonize northern Europe by 2050 and northern America by the end of the century, potentially creating new epidemiological scenarios [5]. This fact allows the categorization of this mosquito species as one of the 100 worst invasive species in the world [6], and together with other species of the genus *Aedes*, it is considered to have the highest economic impact of all invasive organisms [7]. Moreover, *Ae. albopictus* is not only a nuisance due to mosquito bites but is also a well-known vector of several pathogens affecting humans, including viruses such as dengue virus and Zika virus, as well as parasites including *Dirofilaria* [8,9,10]. The introduction of *Ae. albopictus* to new areas could facilitate local transmission of these pathogens in the invaded areas, which is responsible for several outbreaks, potentially impacting the epidemiology of some mosquito-borne diseases [11].

Dengue is the most frequent arboviral disease, with more than one million cases per year since 2019 just in America [12]. In Europe, an increasing number of autochthonous infections have been happening since 2010 [13], where the presence of *Ae. albopictus* has allowed its local transmission [14]. Zika virus, which is also transmitted by *Aedes* mosquitoes, has been responsible for at least three major outbreaks, namely Yap Island in 2007, French Polynesia in 2013, and Brazil in 2016. In America, by 2017, more than 220,000 cases of Zika were confirmed and more than 580,000 cases were suspected [15]. *Aedes aegypti* is considered an important vector of this pathogen [16], but in Europe where this species has a limited distribution range, *Ae. albopictus* may allow Zika transmission [17]. *Dirofilaria immitis* and *Dirofilaria repens* are endemic pathogens in Europe with an increasing prevalence trend in central, northern, and eastern Europe, including countries such as Romania and Greece, potentially favored by climate change and the presence of invasive *Aedes* species, including *Ae. albopictus* [18]. Furthermore, several studies have pointed out the potential relevance of *Ae. albopictus* transmitting other pathogens such as the zoonotic West Nile virus [19]. This virus naturally circulates between mosquitoes and wild birds, but occasionally it can affect humans and horses [20].

These articles support the role of invasive populations of *Ae. albopictus* affecting the local transmission of pathogens of public health relevance. However, studies about how the Asian tiger mosquito interacts with wildlife pathogens are scarce, even though its introduction to new areas could also alter the transmission dynamics of wildlife diseases. One such pathogen is the widespread avian malaria parasite (*Plasmodium* spp.), but the information regarding its interaction with *Ae. albopictus* and the role of this invasive mosquito species as a vector is fragmented. Here, we aim to compile the information available about the relationship established between avian *Plasmodium* and *Ae. albopictus*, to identify the role of this invasive mosquito species as a potential vector of parasites under natural conditions. In addition, we identify the current knowledge gaps on this research topic to propose future research lines.

## 2. Avian Malaria Parasites

Avian *Plasmodium* parasites (Apicomplexa, Haemosporidia) are common blood parasites infecting birds in all continents except Antarctica. Infections by avian malaria parasites have detrimental effects on wild bird individuals and populations. Experimental treatment with the antimalarial primaquine of infected birds lead to an increase in fitness parameters such as clutch size, hatching success, and number of fledgings compared to untreated ones [21]. Furthermore, malaria infection reduces life span and the number and quality of nestlings, probably mediated by a higher telomere shortening [22]. In addition, the introduction of avian malaria parasites in areas with immunologically naïve individuals, such as Hawaii, generates a new epidemiological scenario contributing to the population decline of native avian populations [23]. The high susceptibility of native species to avian malaria, reaching high levels of parasitemia, contributed, at least in part, to the important rate of extinction of endemic avifauna in this area, and also altered the distribution of native birds [24]. Avian *Plasmodium* has been also identified as a potential factor affecting the population decline of common species such as house sparrow, *Passer domesticus,* in UK, where it shows a 71% population decline since 1995 [25]. According to this study, infection intensity of *Plasmodium* was higher in juveniles from declining bird populations and negatively correlated with adult and juvenile survival, finally reducing the recruitment of juveniles in the populations.

Avian *Plasmodium* represents a diverse group including more than 50 morphospecies [26], although the genetic diversity of these parasites is much higher. According to Malavi, the largest database of avian haemosporidian parasites infecting birds [27], more than 1500 different genetic lineages have been described to date (accessed on 11 March 2024). Different morphospecies and genetic lineages can deeply differ in important aspects of their ecology or development, affecting disease dynamics. Namely, they could have different degrees of host specificity, geographic range, seasonal occurrence and effects on hosts (see references in [28]).

The avian *Plasmodium* life cycle consists of a sexual reproduction, which takes place in the mosquitoes, and an asexual development, which happens in the birds [29]. Briefly, an infected mosquito injects sporozoites together with its saliva during the feeding. These sporozoites start the exo-erythrocytic development of *Plasmodium* in the bird tissues, with the first generation of meronts, called cryptozoites, developing in the reticular cells of several organs. These cryptozoites induce the second generation of meronts, called metacryptozoites, in macrophages of many bird organs. These metacryptozoites can infect blood cells, when parasitemia starts and the parasite can be seen in blood smears. They can also infect other organs, receiving the name of phanerozoites, which are responsible for the relapses during the infection. Once in the blood, the parasite starts its development, forming erythrocytic meronts, or macro- and microgametocytes. These gametocytes are infective stages for the mosquito vectors and are acquired by them during a blood meal. In the mosquito midgut, mature gametocytes escape from the erythrocytes, fertilization occurs, and motile ookinetes are formed. They invade the midgut wall and develop into oocysts, which form the sporozoites. When mature, the oocysts rupture, and sporozoites are released, penetrating the hemocoel to reach the salivary glands [29]. Avian *Plasmodium* can be transmitted by mosquitos (Diptera: Culicidae) belonging to several genera, such as *Aedes*, *Anopheles*, *Culex*, and *Culiseta* [29,30]. Although differences exist between parasite and mosquito species [31], the specificity of *Plasmodium* parasites for vector species is generally low, with species such as *Plasmodium relictum* being able to complete its sporogony development in mosquitoes belonging to six different genera and over 20 species [29]. As in the case of birds, infection by avian malaria parasites may have detrimental effects on mosquitoes, for example, in terms of survival rate [32,33], which extensively contribute to vectorial capacity.

The role of mosquitoes as vectors of avian *Plasmodium* parasites has been poorly investigated, at least compared to the number of studies conducted on the interactions between parasites and their bird hosts. Parasite prevalence in wild-caught mosquitoes is frequently low, requiring sampling hundreds or thousands of individuals to obtain reliable epidemiological data. Even when molecular analysis has facilitated the identification of parasites in mosquitoes captured in the wild, experimental procedures are necessary to truly identify the vector species of avian malaria parasites. Such analyses are fairly common with laboratory-reared species but are rare on mosquito individuals captured in the wild [34]. If the mosquito studied is not suitable for parasite development, researchers have to face two main issues: (i) the number of individuals required to confirm the non-development of a parasite in low prevalent species; and (ii) the difficulty in publishing negative results [35].

## 3. Molecular Xenomonitoring of Avian *Plasmodium* in *Aedes albopictus*

Different approaches have been used to explore the role of several mosquito species in the transmission of avian *Plasmodium*, including directly identifying the parasite infective forms (sporozoites) in the mosquito salivary glands and, most commonly, the identification of *Plasmodium* DNA in wild-collected mosquitoes through molecular analysis. The identification of parasite DNA in wild-collected mosquitoes, also known as molecular xenomonitoring, is a common procedure to identify potential vectors of avian *Plasmodium* in different ecosystems. To do that, authors have molecularly tested the presence of parasites in the whole body of mosquitoes, their head or their thorax, where the salivary glands of mosquitoes are located. However, molecular identification of parasite DNA in mosquitoes does not imply vector competence, because DNA from non-infective forms of parasites could be detected in field-collected insects [36]. For example, avian malaria like parasites of the genus *Haemoproteus*, which are not transmitted by mosquitoes, are able to develop until the oocyst stage in mosquitoes, so could be molecularly detected, even though the sporozoites are not found in their salivary gland [37]. Nevertheless, molecular approaches give important insights about the contacts established between mosquitoes and parasite species and lineages, as well as the vector feeding behavior in the wild [38]. Thus, molecular xenomonitoring helps to identify the role of mosquito species in the transmission of avian *Plasmodium* in a particular area.

**Figure 1:**
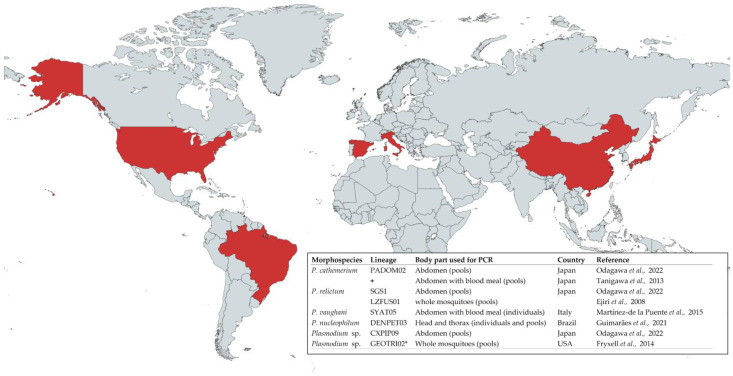
In red, countries where the presence of avian *Plasmodium* have been molecularly tested in wild-caught *Ae. albopictus*. The table shows *Plasmodium* lineages and their corresponding morphospecies, found in *Ae. albopictus* mosquitoes [39,40,44,50,51,53]. * The lineage GEOTRI02 was named according to Malavi after BLAST comparison with the sequence published in GenBank (reference EU328176) by the authors of the study. **+** Authors reported the identified *Plasmodium* lineage as Ts143h, which corresponds to PADOM02 according to Malavi.

This procedure has been repeatedly used in the case of *Ae. albopictus* sampled in different countries in Asia, America, and Europe (Figure 1). As a result, different avian *Plasmodium* lineages corresponding to different morphospecies have been found (Figure 1). Most studies on the interaction between avian malaria parasites and *Ae. albopictus* have been conducted in Asia, mostly in Japan, where a generally low prevalence of avian *Plasmodium* infection was reported in wild *Ae. albopictus*. For instance, *Ae. albopictus* mosquitoes were sampled at different locations in Minami Daito Island, Japan, and only a single mosquito pool out of the 46 tested (including a total of 81 mosquitoes) was positive for the presence of avian *Plasmodium* DNA [39]. Also, in mosquitoes collected in Tsushima Island (Japan), just one fully fed female individual from the 93 *Ae. albopictus* collected was positive for avian *Plasmodium* [40]. Further studies conducted in the zoo of Kanagawa, Japan, revealed a total absence of avian *Plasmodium* in 40 mosquito pools, in a total of 330 specimens of *Ae. Albopictus* tested [41]. This was also the case for mosquitoes captured in Tokyo, where avian *Plasmodium* was not found in 668 mosquitoes grouped in 69 pools [42], and in Niigata, also in Japan, where avian *Plasmodium* was not found in the 13 mosquitoes tested [43]. Furthermore, an interesting long-term study was conducted in Kanagawa (Japan) where mosquito populations were studied during 10 consecutive years in four different localities. The authors of this study identified all the mosquitos captured and explored the presence of avian malaria infection including 5176 individuals of *Ae. albopictus*. *Plasmodium* DNA was only detected in samples collected in 2015 with a minimum infection rate (number of PCR positive sample/number of mosquito collected × 1000) of 4.9 for that year [44]. An additional study performed across China revealed that none of the 806 *Ae. albopictus* captured were positive for avian *Plasmodium* [45].

Studies on *Ae. albopictus* mosquitoes captured in Europe also support a generally low avian *Plasmodium* prevalence of infection. In Barcelona (Spain), none of the 84 mosquito pools tested (including 473 mosquito females) were positive for the presence of avian *Plasmodium* DNA [46]. Similarly, all the 23 mosquito pools of *Ae. albopictus* (including 92 mosquitoes) captured in the southern provinces of Granada and Malaga in Spain were negative for the presence of avian *Plasmodium* DNA [47]. In addition, a recent study conducted in the Lazio region (central Italy) reported a total absence of avian *Plasmodium* in 11 mosquito pools (containing 545 *Ae. albopictus* females) [48]. Similar results have also been found in other regions of the current distribution range of the species, such as America. For example, in Oklahoma (USA), none of the 1343 *Ae. albopictus* mosquitoes captured and grouped in 298 mosquito pools were positive for the presence of avian *Plasmodium* DNA [49]. However, another study in Tennessee, also in the USA, identified 12 positive mosquito pools for *Plasmodium* from a total of 148 *Ae. albopictus* pools tested [50]. The authors of this latter study identified a single parasite lineage in *Ae. albopictus* which was previously found infecting the common yellowthroat (*Geothlypis trichas*) (Figure 1). In South America, one positive *Ae. albopictus* individual (out of 15 tested) for avian *Plasmodium* DNA was also found in São Paulo, Brazil [51], while in other study in the same country, none of the 11 *Ae albopictus* captured was positive for avian malaria parasites [52]. See Figure 1 for a complete list of the parasite species and lineages found in wild-caught *Ae. albopictus*.

Overall, these studies suggest that, although *Ae. albopictus* may occasionally harbor avian *Plasmodium* DNA, the role of this mosquito species in the transmission of avian malaria parasites is likely limited. This clearly contrasts with the pattern found for other mosquito species captured in the same areas [41,42,44,46,47,48,49]. This is the case of, for example, mosquito species belonging to the *Culex pipiens* complex, where avian *Plasmodium* DNA is frequently found, suggesting a key role of this mosquito species in the local transmission of avian malaria parasites under natural conditions [30,53,54]. In other words, these studies support the idea that, although local transmission of avian malaria parasites occurs in these areas, the relevance of *Ae. albopictus* for the circulation of avian *Plasmodium* may be low in comparison with other species, including *Cx. pipiens*.

The overall low prevalence of infection by avian *Plasmodium* found in wild-caught *Ae. albopictus* mosquitoes has been traditionally explained based on the mammal-biased blood-feeding pattern of this species. *Aedes albopictus* is able to feed on blood from a range of vertebrates, including mammals, birds, reptiles, and fish [55]. However, mammals, including humans, are frequently reported as the main blood-feeding source of *Ae. albopictus* mosquito females [55], with a low frequency of mosquitoes of this species feeding on *Plasmodium*-infected birds [53,56]. Nevertheless, other potential factors including the vector–parasite compatibility could also explain, at least in part, the results found.

## 4. Experimental Studies on the *Aedes albopictus* Competence for Avian *Plasmodium*

Although molecular xenomonitoring studies offer valuable insights into the significance of the mosquito species involved in the transmission of avian parasites in the wild, the presence of avian *Plasmodium* DNA in mosquitos does not necessarily imply vector competence [36]. Vector competence is the mosquito’s capability to acquire, support the replication and/or development, and transmit the pathogen to a susceptible host, usually indicated by the presence of parasite sporozoites (avian *Plasmodium* infective stage) in mosquito salivary glands [57]. However, avian *Plasmodium* development in mosquitoes could be abortive [58], which makes the identification of the different life stages of parasites crucial for analyses of vector competence. Therefore, experimental approaches to identify the infective forms of parasites in mosquito salivary glands or their saliva, or even better, the infection of uninfected vertebrate hosts by the bites of infected mosquitoes, are essential to identify the vectors of avian malaria parasites which could contribute to avian malaria circulation in the wild.

Various studies, most of them conducted during the previous century, have used experimental approaches to identify the development of different morphospecies of avian *Plasmodium* in *Ae. albopictus* females [30,59] (Table 1). The vector competence for avian malaria of *Ae. albopictus* was previously reviewed by Huff in 1965 [59]. This author reported that (i) no oocysts were found in *Ae. albopictus* mosquitoes for the species *Plasmodium cathemerium, Plasmodium circumflexum, Plasmodium vaughani*, and *Plasmodium hexamerium*; (ii) oocysts and efficient transmission by *Ae. albopictus* bites were found for *Plasmodium fallax*; and (iii) oocysts, sporozoites and efficient transmission by *Ae. albopictus* bites were found for *Plasmodium gallinaceum* and *Plasmodium lophurae* (Table 1). Furthermore, Weathersby [60] injected sporozoites, oocysts or gametocytes of *P. fallax* into mosquito hemocoel and found a successful parasite development in more than 10% of the *Ae. albopictus* analyzed.

More recently, an experimental study analyzing the development of different isolates of *Plasmodium elongatum* in several mosquito species revealed that this species was unable to reach oocyst or sporozoite stages in any of the 40 *Ae. albopictus* tested [61]. Further studies have been conducted using *P. relictum*. Although Van Ripper III did not find *P. relictum* sporozoites in any of the three *Ae. albopictus* analyzed [62], a more recent study using a higher sample size (n > 90) found both oocysts and sporozoites in *Ae. albopictus* females from Hawaii [63]. However, the prevalence of parasite infection was very low in the examined mosquitoes, with parasites being found in only 1% of the tested individuals and with a low intensity of infection. This is especially relevant when comparing the results found for *Ae. albopictus* with those obtained for *Culex quinquefasciatus* in the same study. In the latter species, oocysts and sporozoites were found in more than 86% of the tested individuals and with higher intensities of infection [63]. Additionally, O’Donnell and Armbruster confirmed the susceptibility of different lines of *Ae. albopictus* for *P. gallinaceum* [64]. These authors identified *Ae. albopictus* as a highly competent mosquito vector showing parasite oocysts and sporozoites in this mosquito species and confirming true transmission to uninfected birds after being bitten by infected *Ae. albopictus* females [65].

**Table 1:** Avian *Plasmodium* morphospecies experimentally tested for their development in *Aedes albopictus* mosquito females.

*Plasmodium* Morphospecies	Most Advanced Developmental Stage Found	Efficient Transmission	References
*P. gallinaceum*	Sporozoite	Yes	[59,64,65,66,67,68,69,70,71,72,73]
*P. fallax*	Sporozoite	Yes	[59,60,74]
*P. lophurae*	Sporozoite	Yes	[59,75,76,77]
*P. cathemerium*	No oocyst detected	No	[78]
*P. circumflexum*	No oocyst detected	No	[59]
*P. vaughani*	No oocyst detected	No	[59]
*P. hexamerium*	No oocyst detected	No	[59]
*P. relictum*	Sporozoite	No	[63]
*P. elongatum*	No oocyst or sporozoites detected	No	[61]

Therefore, according to the available literature, the avian malaria morphospecies known as able to complete their development in *Ae. albopictus* include *P. fallax*, *P. lophurae, P. gallinaceum*, and *P. relictum*. *Plasmodium fallax*, a parasite species that was reported infecting birds belonging to Falconiformes, Strigiformes, Galliformes, and Passeriformes [29], seems to develop relatively well in *Ae. albopictus*, even when compared to *Cx. quinquefasciatus* [74]. This parasite species was initially isolated from the helmeted guineafowl (*Numida meleagris*) in Uganda [74], and several studies have been performed with laboratory-maintained parasites in the previous century [79,80,81]. This parasite species was also found in naturally infected kestrels (*Falco tinnunculus*) in the Cape Verde archipelago [82]. *Plasmodium lophurae* has been found in birds belonging to Columbiformes and Galliformes, but was only isolated once from its natural host, the Bornean crested fireback (*Lophura igniti igniti*) [29]. Since then, only a few studies have explored their development in mosquitoes because the parasite lost the ability to produce gametocytes after several blood passages in the laboratory [29]. Furthermore, parasite development only occurred in mosquitoes under specific laboratory conditions. *Plasmodium gallinaceum* has been found in Galliformes, typically infecting domestic chickens (*Gallus gallus domesticus*). This parasite species has been extensively studied and has a wide range of vectors, including *Ae. albopictus*, which is highly susceptible to the infections by this parasite [29]. Finally, the widespread species *P. relictum,* one of the best-studied species of avian malaria parasites, is able to complete its development in more than 20 mosquito species, including *Ae. albopictus*. However, in comparison with other mosquito species of the *Culex* genus, *Ae. albopictus* seems to be less susceptible to infection [83]. *Plasmodium relictum* is considered a generalist parasite of birds [84].

In summary, only some of the examined *Plasmodium* morphospecies are able to complete their development in *Ae. albopictus*. Therefore, the susceptibility of *Ae. albopictus* mosquitoes depends on the parasite species, with vector–parasite compatibility being an important factor that modulates *Plasmodium* epidemiology in each scenario. However, studies of parasite development are mostly explored under unnatural laboratory conditions with lab-reared mosquito colonies and only with a fraction of known *Plasmodium* species, which might not fully represent the real scenario occurring in the wild.

## 5. The Role of Other Invasive *Aedes* Mosquitoes as Avian Malaria Vectors

Although *Ae. albopictus* is considered a major invasive species, other species within the *Aedes* genus are also highly invasive. For example, in the case of Europe, established populations of *Aedes koreicus* and *Aedes japonicus* have been reported in different countries [85]. As with *Ae. albopictus*, mammals dominate the diet of these species, including humans [55]. However, at least in the case of *Ae. japonicus*, birds are occasionally found in its diet, including species such as *G. domesticus*, *Turdus merula*, *P. domesticus*, *Spheniscus humboldti* and *Rhea pennata* [55], suggesting that this mosquito species could interact with avian malaria parasites infecting these avian hosts. However, although the presence of avian *Plasmodium* has been poorly investigated in *Ae. japonicus*, current information suggests that this mosquito species may not be a major avian malaria vector in nature due to its low prevalence [44,86,87]. Particularly, in the aforementioned long-term study performed in Kanagawa by Odagawa et al. [44], of the 197 *Ae. japonicus* mosquitoes captured in Japan, none were positive for avian malaria parasites. Similarly, avian malaria parasites were not found in a single engorged *Ae. koreicus* mosquito captured in Hungary, with all of them containing human blood-meals [88]. Other introduced species of the *Aedes* genus have been also tested for the presence of avian malaria parasites in their current distribution range. That is the case of *Aedes notoscriptus* in New Zealand, for which a minimum infection rate of avian *Plasmodium* was 1.79% [89]. The parasite lineages identified in the thoraxes of this mosquito species include LINN1 (*Plasmodium matutinum*), GRW6 (*P. elongatum*) and SYAT05 (*P. vaughani*) [89]. Nevertheless, the information on avian malaria in these mosquito species has been seldom investigated and further research is necessary in order to effectively address questions about its vectorial role.

Contrary to the case of these *Aedes* species, the role of the invasive yellow fever mosquito *Ae. aegypti* as vector of different avian malaria parasites has been deeply addressed, especially in experimental studies. According to the reviews by Huff et al. [59] and Santiago-Alarcon et al. [30], avian malaria parasites including *P. gallinaceum* and *P. relictum* are able to complete their development until the sporozoite stage or be transmitted in this mosquito species, and *P. cathemerium* is able to develop until oocyst stage. However, this is not the case of other species, including *P. elongatum, Plasmodium heroni, P. hexamerium, Plasmodium juxtanucleare,* and *P. vaughani,* as no oocysts were observed for these species. Further studies on the development of *P. gallinaceum* in *Ae. aegypti* have confirmed its development [65,90], while an inability to develop in *Ae. aegypti* has been shown for *P. delichoni* [58]. Positive amplification of a lineage of the closely related *Haemoproteus* parasite was found in *Ae. aegypti* from New Caledonia [91], which may be due to the presence of DNA from abortive stages of the parasite [37]. Similarly to the results concerning *Ae. albopictus,* although the development of some avian *Plasmodium* species may be possible in *Ae. aegypti*, avian blood meals in *Ae. aegypti* are uncommon [33], likely limiting the contact rates between this mosquito species and *Plasmodium* parasite-infected birds under natural conditions.

## 6. Conclusions

The invasive mosquito *Ae. albopictus* drastically increased its distribution range during the last decades and it is currently reaching areas of higher latitudes in Europe. This invasive distribution pattern could alter the epidemiological scenarios of mosquito-borne pathogens in the areas where it is currently established. Different species of avian *Plasmodium* can complete their development in *Ae. albopictus* mosquito females, at least for a handful of avian malaria species tested so far. However, most studies targeting *Ae. albopictus* vector competence were conducted in the previous century with laboratory-reared mosquitoes and limited *Plasmodium* species and lineages. Furthermore, the prevalence of avian *Plasmodium* in wild *Ae. albopictus* mosquitoes is extremely low, which suggests that the relevance of this invasive mosquito species for the local transmission of avian malaria parasites under natural conditions may be low. However, we have noticed that most of the negative results found for this species are presented as secondary results in the articles, making it difficult to obtain a conclusion of the overall role of *Ae. albopictus* as a vector of avian *Plasmodium*. Factors including the mammal-biased blood-feeding habit of this mosquito species and its reduced competence for the development of avian *Plasmodium*, at least for some of the parasite species tested, should be responsible for the low relevance of *Ae. albopictus* as vectors of avian malaria parasites in natural environments.

## 7. Future Directions

Studies on the role of different mosquito species in the transmission of avian malaria parasites have been significantly increasing during the last years. Despite that, current knowledge of the mosquito species involved in the transmission of avian malaria parasites in the wild is still limited, at least compared to the case of studies conducted in birds. Based on the high diversity of avian *Plasmodium* morphospecies and, especially, parasite lineages found infecting birds in nature, studies exploring the compatibility of *Ae. albopictus* for other avian *Plasmodium* species should be addressed. This is also the case of other invasive *Aedes* mosquitoes, which are currently increasing their distribution range, potentially affecting the avian *Plasmodium* dynamics in the introduced areas. These studies should also consider other organisms potentially affecting the interaction between mosquitoes and the pathogens that are able to transmit, including mosquito microbiota. This biotic factor has been recently proven to interact with pathogens through several mechanisms, significantly affecting its vector competence [92], including studies with *Ae. albopictus* [93].

## Data Availability

Not applicable.
